# Validity of the prognostication tool PREDICT version 2.2 in Japanese breast cancer patients

**DOI:** 10.1002/cam4.3713

**Published:** 2021-01-15

**Authors:** Karen Zaguirre, Masaya Kai, Makoto Kubo, Mai Yamada, Kanako Kurata, Hitomi Kawaji, Kazuhisa Kaneshiro, Yurina Harada, Saori Hayashi, Akiko Shimazaki, Takafumi Morisaki, Hitomi Mori, Yoshinao Oda, Sanmei Chen, Taiki Moriyama, Shuji Shimizu, Masafumi Nakamura

**Affiliations:** ^1^ Department of Surgery and Oncology Graduate School of Medical Sciences Kyushu University Fukuoka Japan; ^2^ Institute of Surgery St. Luke’s Medical Center Quezon City Philippines; ^3^ Department of Anatomic Pathology Graduate School of Medical Sciences Kyushu University Fukuoka Japan; ^4^ Department of Epidemiology and Public Health Graduate School of Medical Sciences Kyushu University Fukuoka Japan; ^5^ International Medical Department Kyushu University Hospital Fukuoka Japan

**Keywords:** breast cancer, prognosis, survival, women's cancer

## Abstract

**Introduction:**

PREDICT is a prognostication tool that calculates the potential benefit of various postsurgical treatments on the overall survival (OS) of patients with nonmetastatic invasive breast cancer. Once patient, tumor, and treatment details have been entered, the tool will show the estimated 5‐, 10‐, and 15‐year OS outcomes, both with and without adjuvant therapies. This study aimed to conduct an external validation of the prognostication tool PREDICT version 2.2 by evaluating its predictive accuracy of the 5‐ and 10‐year OS outcomes among female patients with nonmetastatic invasive breast cancer in Japan.

**Methods:**

All female patients diagnosed from 2001 to 2013 with unilateral, nonmetastatic, invasive breast cancer and had undergone surgical treatment at Kyushu University Hospital, Fukuoka, Japan, were selected. Observed and predicted 5‐ and 10‐year OS rates were analyzed for the validation population and the subgroups. Calibration and discriminatory accuracy were assessed using Chi‐squared goodness‐of‐fit test and area under the receiver operating characteristic curve (AUC).

**Results:**

A total of 636 eligible cases were selected from 1, 213 records. Predicted and observed OS differed by 0.9% (*p* = 0.322) for 5‐year OS, and 2.4% (*p* = 0.086) for 10‐year OS. Discriminatory accuracy results for 5‐year (AUC = 0.707) and 10‐year (AUC = 0.707) OS were fairly well.

**Conclusion:**

PREDICT tool accurately estimated the 5‐ and 10‐year OS in the overall Japanese study population. However, caution should be used for interpretation of the 5‐year OS outcomes in patients that are ≥65 years old, and also for the 10‐year OS outcomes in patients that are ≥65 years old, those with histologic grade 3 and Luminal A tumors, and in those considering ETx or no systemic treatment.

## Graphical abstract

This study conducted an external validation of the online PREDICT tool version 2.2 by studying its predictive accuracy of the 5‐ and 10‐year overall survival (OS) in a population of breast cancer patients in Japan. Based on analysis of 636 eligible patients, the differences of the predicted and observed 5‐ and 10‐year OS were not statistically significant and the discriminatory accuracy results were fairly well. Hence, we can say that PREDICT tool accurately estimated the 5‐ and 10‐year overall survival in the Japanese validation population.

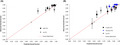

## 1 INTRODUCTION

Breast cancer is the leading cancer in women all over the world. It is also the most frequent cause of mortality from cancer in women regardless of race or ethnicity. In 2018, over 2 million newly diagnosed cases of breast cancer and more than 600,000 breast cancer‐specific mortalities were reported worldwide.^1^ In Japan, a total of 92,253 new breast cancer cases and 14,285 deaths were recorded in 2017.^2^

Adjuvant systemic treatment is a systemic therapy given after surgery to stop or prevent micrometastasis. It has been proven to reduce the risk of recurrence and breast cancer mortality.^3^, ^4^ Accurate appraisal of prognosis and prospective benefit from additional postsurgical systemic therapy could help minimize undertreatment and overtreatment. This can help both physicians and patients in choosing the best treatment option that will provide the optimal therapeutic benefit while reducing the side effects and maintaining the quality of life.^5^, ^6^ Several prediction models have been created to assist in deciding regarding which adjuvant systemic therapy is most suitable for the patient depending on the patient and tumor characteristics, such as Adjuvant! Online and PREDICT. In comparison with Adjuvant! Online, PREDICT incorporates other factors such as mode of detection, Ki67 status, and HER2 status, however, it does not include patient's comorbidities.^7^ Validation studies in Asian patients have shown that Adjuvant! Online was overoptimistic in predicting overall survival.^8^ Another study done in Southeast Asian patients have shown that PREDICT was accurate in most subgroups of patients, but was overoptimistic in young patients (<40 years), and in those receiving neoadjuvant chemotherapy.^7^ Adjuvant! Online is a well‐known and widely used clinical prognostication model, however, it has been unavailable for quite sometime.^9^, ^10^ Currently, PREDICT is the only free online prediction tool that has been advocated by the American Joint Committee on Cancer.^11^

PREDICT is a prognostication tool that shows how various postsurgical treatments can potentially improve the overall survival (OS) of patients with nonmetastatic invasive breast cancer. Once patient, tumor, and treatment details have been entered, the tool will show the estimated 5‐, 10‐, and 15‐year OS rates, with and without postoperative treatments (systemic chemotherapy, hormonal therapy, trastuzumab, and bisphosphonates). Results are displayed in visual and textual formats.^12^

PREDICT was a collaboration between the Cambridge Breast Unit, University of Cambridge Department of Oncology, and the UK's Eastern Cancer Information and Registration Centre. The original model was based from the cancer databank information involving 5694 women treated in East Anglia from 1999 to 2003, and was validated using data of more than 5000 patients with breast cancer from the West Midlands Cancer Intelligence Unit.^12^, ^13^ The estimated benefits from the treatments were based on the Early Breast Cancer Trialists’ Collaborative Group meta‐analyses of clinical trials.^4^

The pioneer model (version 1) of PREDICT was made available online in 2010 and the tool has become more well‐known thereafter.^13^, ^14^ Several adjustments and updates have been made afterwards that helped improve the tool's OS estimates.^15^ The initial upgrade in 2011, version 1.1, incorporated human epidermal growth factor 2 (HER2) status, and included the estimated treatment effect if ever trastuzumab will be given.^12^, ^16^ In version 1.2, the proliferation marker Ki‐67 was added as a prognostic parameter.^17^ In 2017, model re‐fitting (version 2.0) was done, with improvement on the age at diagnosis, exact size of the tumor in millimeters, and the number of positive lymph nodes.^12^, ^18^ Addition of bisphosphonates as treatment option and addition of 15‐year outcomes were done for version 2.1. In the latest model, version 2.2, option for extended hormonal therapy for an additional 5 years was added.^12^

PREDICT version 1 was validated in several studies from various countries, such as Canada,^19^ the Netherlands,^9^, ^20^ Malaysia,^7^ and the United Kingdom.^17^, ^21^ However, the validity of PREDICT has not yet been verified on the general Japanese breast cancer patients.

This study aimed to conduct an external validation of the prognostication tool PREDICT version 2.2 by evaluating its predictive accuracy of the 5‐ and 10‐year OS outcomes among female patients with nonmetastatic invasive breast cancer in Japan.

## 2 METHODS

### 2.1 Patient selection

Data were retrieved from a hospital registry of consecutive patients diagnosed with breast cancer, identified through the breast cancer databank of Kyushu University Hospital (KUH), Fukuoka, Japan. All female patients who were diagnosed from 2001 to 2013 with unilateral, nonmetastatic, invasive breast cancer and who underwent corresponding surgery (mastectomy or breast‐conserving surgery), were selected. Patients with unknown age, tumor size, tumor grade, estrogen receptor (ER) status, number of positive lymph nodes, and adjuvant treatment, were excluded because these are required variables in PREDICT, and absent values for such will not be accepted. Patients with age <25 and >80 years were excluded as well, since PREDICT only accepts age within that range. Data regarding follow‐up of each patient, including date of last follow‐up and date of death were also acquired from the same breast cancer databank, and patients with unknown follow‐up time were excluded.

The treatment approach for the patients were based on the National Comprehensive Cancer Network (NCCN) clinical practice guidelines for breast cancer,^22^ the St. Gallen International Breast Cancer Conference consensus,^23^, ^24^, ^25^, ^26^ and the Japanese Breast Cancer Society clinical practice guidelines.^27^ This research complied to the Declaration of Helsinki codes and was accepted by the Institutional Review Board of Kyushu University Hospital (No. 30–230).

### 2.2 Follow‐up

The total duration of inclusion of each patient in the study was computed from the date of surgical treatment for breast cancer until death, or until censored at the completion of follow‐up period (October 1, 2019), or when 10 years of follow‐up was reached.

### 2.3 Immunohistochemistry (IHC) staining

The different tumor subtypes were determined using IHC staining of the resected specimens. The tissue specimens that were used for IHC were immediately fixed within 1 h of surgical removal in 10% neutral‐buffered formalin for 6 to 72 h. ER‐positive and progesterone receptor (PR)‐positive specimens were interpreted as having ≥1% of tumor cells that stained positive for ER or PR, respectively.^28^ Tissue specimens were labeled as HER2‐positive when the IHC staining obtains a score of 3+ based on the standard criteria, or when the score is 2+ and the fluorescence in situ hybridization shows HER2 gene amplification.^29^, ^30^ Tissue specimens were labeled as luminal B when Ki‐67 status was high (> 20%) or PR status was low (< 20%) in an ER‐positive disease.

### 2.4 PREDICT scores

Predicted OS rates were obtained by manually entering the necessary details for each patient in the PREDICT tool (version 2.2), with blinding to patient outcomes. If there is a missing information on any of the nonmandatory variables, such as the menopausal status, HER2 status, Ki‐67 status, mode of detection, and presence of lymph node micrometastasis (if only one positive lymph node was harvested), patients were not excluded in the study, but the “unknown” option was selected instead. Ki‐67 status was not routinely requested until late 2010, hence, for all cases before that, the “unknown” tab was selected for this variable.

The resulting predicted 5‐ and 10‐year OS outcomes based on the actual therapy given to each patient was documented. Since the prognosticator variables were manually entered in PREDICT, the results are prone to encoding error. Hence, all the PREDICT scores were calculated three times to ensure accuracy of the obtained data.

### 2.5 Statistical analyses

All statistical analyses were done in IBM SPSS Statistics version 25. A *p*‐value of ≤ 0.05 was defined as statistically significant.

The Kaplan–Meier method was used for the survival analysis. The 5‐ and 10‐year observed OS rates for the whole validation population and the subgroups were based from the survival estimates on the Kaplan–Meier curve. The median values were used for the predicted 5‐ and 10‐year OS outcomes for the whole population and for the subgroups.^7^ To evaluate the goodness‐of‐fit of the tool, the observed and predicted events for the entire study population as well as for all subgroups were analyzed using Chi‐squared test. In line with the Dutch validation study, an *a priori* assumption was set, which states that PREDICT tool correctly prognosticated the OS rates if the difference of the predicted and observed outcomes is not more than 5%, since a difference more than this value will be considered as clinically relevant.^6^

Calibration of the model was assessed using Chi‐squared test and by making a calibration plot for the survival rates. The entire study population was initially binned into quintiles of the predicted survival rates. A calibration plot was then made showing the observed 5‐ and 10‐year OS outcomes per quintile, against the median of the predicted OS outcomes per quintile.^7^, ^16^ To further evaluate the effect of endocrine therapy (ETx) on OS, model calibration was stratified into the presence and duration of ETx given.

The discriminatory performance of PREDICT was assessed by using receiver operator characteristic (ROC) curve analysis, and by computing for the area under the ROC (AUC) for both the 5‐ and 10‐year OS. A plot was made comparing the number of patients who were alive at the duration of study and were prognosticated accurately (sensitivity), against the number of patients who were deceased but were prognosticated to be alive (1‐specificity). The AUC was utilized to measure the discriminatory accuracy of the tool, and can be interpreted as the probability that patients were accurately prognosticated to be alive or deceased at 5 and 10 years. An AUC value lies between 0.5 and 1, wherein a value of 0.5 suggests that the model has no capacity for discrimination, and a value of 1.0 suggests perfect discrimination.^6^, ^7^

## 3 RESULTS

**FIGURE 1:**
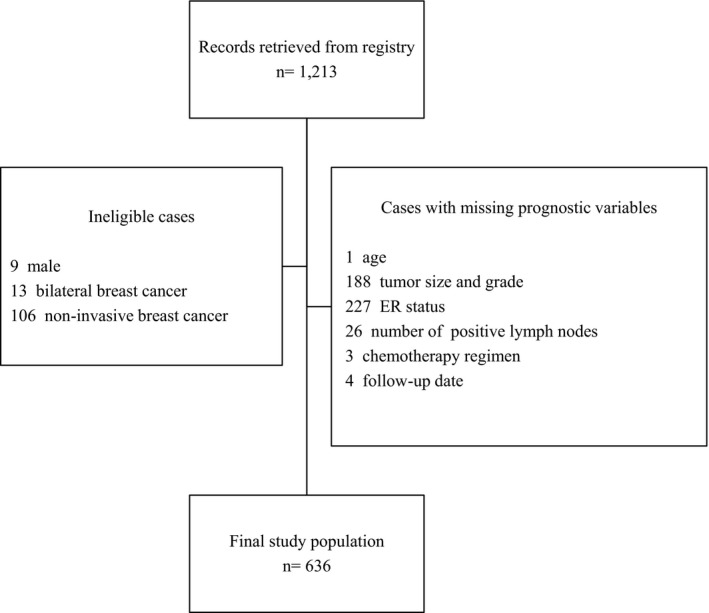
Sample selection flow diagram

A total of 1213 patients diagnosed with breast cancer who had undergone surgical treatment from 2001 to 2013 were identified. Patients who are male (*n* = 9), those with bilateral breast cancer (*n* = 13), and those with noninvasive breast cancer (*n* = 106) were excluded since the data on which PREDICT was based did not include information on the presence of these characteristics. Patients with unknown age (*n* = 1), tumor size and grade (*n* = 188), ER status (*n* = 227), number of positive lymph nodes (*n* = 26), chemotherapy regimen (*n* = 3), and follow‐up date (*n* = 4) were also excluded because these are mandatory variables in PREDICT, and absent values for such will not be accepted by the tool. After all the exclusions, a total of 636 patients remained in the study population (Figure 1).

Observed and predicted 5‐ and 10‐year OS outcomes of the whole population and of the subgroups are presented in Table 1. The median age at diagnosis was 57 years. Most patients were symptomatic at presentation (64%), and only a few were detected through screening (35.7%). Majority of the patients presented with ≤2 cm tumors (64.3%), histologic grade 1 tumor (66%), no lymph node metastasis (70.1%), positive ER status (82.5%), negative HER2 status (74.8%), and Luminal A molecular subtype (47.8%) tumor. More than half of the population underwent breast‐conserving surgery (56.1%) and received ETx only (50.6%) as adjuvant systemic treatment. Majority of those who received ETx discontinued use after 5 years (64%).

**TABLE 1:** Observed and predicted 5‐ and 10‐year overall survival by patient, tumor, and treatment‐related characteristics

Patients	*n* (%)	5‐Year overall survival				10‐Year overall survival			
		Predicted	Observed (s.e)	Difference	*p*‐value	Predicted	Observed (s.e)	Difference	*p*‐value
All Patients	636 (100)	93.7	94.6 (1.0)	−0.9	0.322	86	88.4 (1.7)	−2.4	0.086
Age (years)									
<40	36 (5.7)	97.1	96.8 (3.2)	0.3	0.965	94	93.2 (4.7)	0.8	0.911
40–64.9	408 (64.1)	95.7	94.5 (1.2)	1.2	0.277	90.3	89.4 (2.0)	0.9	0.567
≥65	192 (30.2)	87.4	94.1 (1.9)	−6.7	**0.004**	72.9	84.3 (3.9)	−11.4	**<.001**
Detection									
Screening	227 (35.7)	95.9	98 (1.0)	−2.1	0.146	89.6	93.3 (2.3)	−3.7	0.061
Symptomatic	407 (64)	92.8	92.6 (1.4)	0.2	0.894	84	85.7 (2.3)	−1.7	0.336
Unknown	2 (3)	98	—	—	—	95.2	—	—	—
Tumor size (cm)									
≤2	409 (64.3)	95.3	95.8 (1.1)	−0.5	0.603	89.1	91.6 (1.8)	−2.5	0.093
2.1–5	193 (30.3)	91.5	94.6 (1.7)	−3.1	0.098	82.7	86.2 (3.3)	−3.5	0.224
>5	34 (5.4)	85.4	77.7 (8.2)	7.7	0.140	68.9	54.4 (1.7)	14.5	0.062
Tumor grade									
1	420 (66)	95.55	96.9 (0.9)	−1.35	0.192	89.5	91.9 (1.7)	−2.4	0.108
2	89 (14)	91.5	94 (2.6)	−2.5	0.330	84.9	82.1 (6.2)	2.8	0.448
3	127 (20)	83.4	87.4 (3.2)	−4	0.226	70.9	81.5 (4.4)	−10.6	**0.01**
Positive nodes (*n*)									
0	446 (70.1)	94.4	95.3 (1.1)	−0.9	0.413	88.1	91.6 (1.8)	−3.5	**0.026**
1–3	144 (22.6)	93.3	94.4 (2.1)	−1.1	0.583	85.4	88.9 (3.3)	−3.5	0.236
4–9	27 (4.2)	80.8	91 (6.1)	−10.2	0.120	64.8	60.7 (12.4)	4.1	0.547
>9	19 (3)	79.8	75 (11)	4.8	0.507	61.5	62.5 (14.7)	−1	0.882
ER status									
Negative	111 (17.5)	82.1	85.9 (3.5)	−3.8	0.338	71.4	77 (5.5)	−5.6	0.207
Positive	525 (82.5)	94.9	96.4 (0.9)	−1.5	0.123	88.8	90.9 (1.7)	−2.1	0.135
HER2 status									
Negative	476 (74.8)	93.95	95.4 (1.0)	−1.45	0.205	87.4	90 (1.8)	−2.6	0.098
Positive	91 (14.3)	87.1	89.6 (3.5)	−2.5	0.561	75.8	81.7 (5.4)	−5.9	0.219
Unknown	69 (10.9)	95.1	95.2 (2.7)	−0.1	0.832	87.8	87.9 (4.8)	−0.1	0.878
Ki67 status									
Negative	82 (12.9)	95.7	—	—		89.3	—	—	
Positive	105 (16.5)	90.7	95.8 (2.4)	−5.1	0.053	81.6	78.7 (12.7)	2.9	0.5
Unknown	449 (70.6)	94	93.3 (1.2)	0.7	0.543	86.8	87.5 (1.9)	−0.7	0.649
Molecular Subtype									
Luminal A	304 (47.8)	95.15	98 (0.9)	−2.85	**0.021**	89.05	95.1 (1.8)	−6.05	**0.001**
Luminal B	120 (18.9)	93.9	95.5 (2)	−1.6	0.376	87.4	83.4 (4.7)	4	0.179
HER2+	89 (14)	87.2	89.4 (3.6)	−2.2	0.448	75.8	81.5 (5.4)	−5.7	0.239
Triple‐negative	49 (7.7)	87.3	78.8 (6.3)	8.5	0.105	78.9	74.1 (7.5)	4.8	0.352
Unknown	74 (11.6)	94.55	95.6 (2.5)	−1.05	0.608	86.4	88.4 (4.6)	−2	0.718
Surgery									
Breast‐conserving surgery	357 (56.1)	95.1	97.1 (0.9)	−2	0.066	88.7	94 (1.7)	−5.3	**0.001**
Mastectomy	265 (41.7)	91.5	91.2 (1.9)	0.3	0.883	82.5	80.8 (3.3)	1.7	0.816
Unknown	14 (2.2)	96.1	91.7 (0.8)	4.4	0.531	89.75	91.7 (0.8)	−1.95	0.698
Systemic therapy									
Both	156 (24.5)	94.1	94.4 (1.9)	−0.3	0.945	88.1	85.4 (3.4)	2.7	0.273
CTx only	90 (14.2)	84.65	84 (4.1)	0.65	0.946	75.9	75.9 (5.9)	0	0.939
ETx only	322 (50.6)	95.3	97.8 (0.9)	−2.5	**0.032**	89	94.8 (1.7)	−5.8	**0.001**
None	68 (10.7)	88.95	93.8 (3.5)	−4.85	0.172	75.5	86.4 (6.0)	−10.9	**0.031**
ETx									
No ETx	158 (24.8)	86.5	87.5 (2.9)	−1	0.757	75.55	79.9 (4.3)	−4.3	0.217
With ETx	478 (75.2)	94.95	96.6 (0.09)	−1.6	0.082	—	—	—	—
a. 5 years only ETx	306 (64)	—	—	—	—	85.35	85.2 (3)	0.2	0.992
b. 10 years ETx	172 (36)	—	—	—	—	91.9	98.1 (1.3)	−6.2	**0.002**

Abbrevations: CTx, chemotherapy; ETx, endocrine therapy.

Bold indicates *p* < 0.05.

PREDICT accurately prognosticated the overall short‐term survival of the study population. The difference between the observed 5‐year OS (94.6%) and the predicted 5‐year OS (93.7%) was only 0.9%, *p* = 0.322, which was not statistically significant. The largest difference was noted in the 4–9 positive lymph nodes subgroup, the OS was underestimated by 10.2% (*p* = 0.12), however, the difference was also not statistically significant. The 5‐year OS was significantly underestimated in patients that are ≥65 years old (6.7%, *p* = 0.004), those with Luminal A subtype tumors (2.85%, *p* = 0.021), and in those who received ETx only (2.5%, *p* = 0.032) as adjuvant systemic therapy. However, only the ≥65 years old subgroup had a difference over 5% (Table 1).

The 10‐year OS outcomes were predicted less well as compared to the 5‐year OS outcomes. The predicted 10‐year survival was 86% and the observed 10‐year survival was 88.4%. PREDICT slightly underestimated the overall long‐term survival by 2.4% (*p* = 0.086). The largest difference was in the >5 cm tumor size subgroup, which was overestimated by 14.5% (*p* = 0.062), but was not statistically significant. Significant differences were mostly underestimations observed in the following groups: ≥65 years old (11.4%, *p*=<0.001), tumor grade 3 (10.6%, *p* = 0.01), no positive lymph node (3.5%, *p* = 0.026), Luminal A subtype (6.05%, *p* = 0.001), breast‐conserving surgery (5.3%, *p* = 0.001), no systemic therapy (10.9%, *p* = 0.031), ETx only (5.8%, *p* = 0.001), and 10 years ETx (6.2%, *p* = 0.002). However, the no positive lymph node subgroup had less than 5% difference.

**FIGURE 2:**
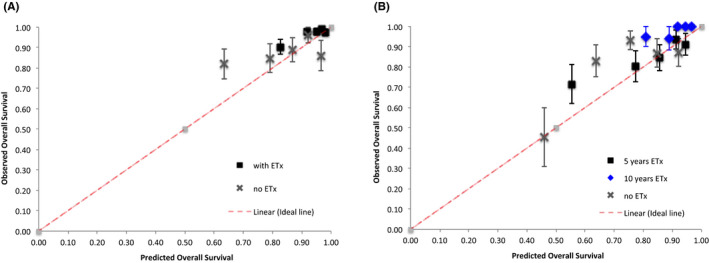
Calibration plot of observed vs. predicted (A) 5‐year and (B) 10‐year overall survival with and without endocrine therapy

A total of 158 (24.8%) patients did not receive ETx and 478 (75.2%) patients received ETx. Out of those who received ETx, 306 patients (64%) had ETx only for ≤5 years, and 172 patients (36%) extended it up to 10 years. Figure 2 shows that the calibration of 5‐year OS versus 10‐year OS was accurate for the higher quintiles of PREDICT score and less accurate for the lower quintiles. The use of ETx and extending it up to 10 years was associated with more accurately predicted OS rates. Statistical analysis revealed that the 5‐year OS (*p* = 0.322) and the 10‐year OS (*p* = 0.086) were not significantly different from the perfect line (x = y).

**FIGURE 3:**
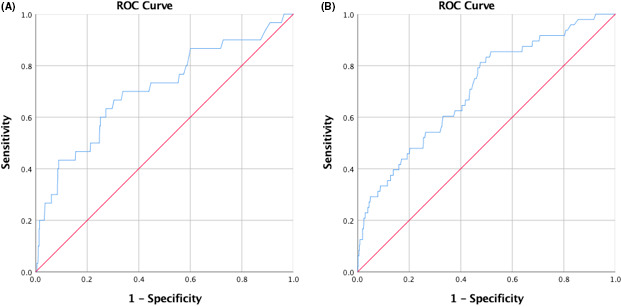
Discriminatory accuracy of (A) 5‐year and (B) 10‐year overall survival

The ROC analysis showed that the tool discriminated fairly well, with an AUC of 0.707 (95% CI: 0.60–0.81) for the 5‐year OS, and 0.707 (95% CI: 0.63–0.78) for the 10‐year OS (Figure 3).

## 4 DISCUSSION

This study shows that PREDICT version 2.2 can accurately prognosticate the 5‐ and 10‐year OS in the whole study population and in several subgroups. The 5‐year OS outcomes were prognosticated accurately, except for the ≥65 years old subgroup, wherein the OS was underestimated by 6.7%. The 5‐year OS difference for Luminal A subtype and ETx only subgroups were below 5%, but were statistically significant and even increased for the 10‐year OS. The 10‐year OS outcomes were predicted quite well, although significant underestimations were observed in the subgroups of ≥65 years old, tumor grade 3, Luminal A subtype, breast‐conserving surgery, ETx only, no systemic therapy, and 10 years ETx.

The discrepancies may be attributed primarily to the differences between the study population (Japan) and the population wherein PREDICT tool was based (United Kingdom). In 2018, the life expectancy of females in Japan was 87 years, while in the United Kingdom it was 83 years.^31^ The higher life expectancy of the Japanese population could have contributed to the underestimation of PREDICT in patients ≥65 years old. The higher observed survival may also be due to the high number of censored data. Only 48 patients reached the event (death), and the other 588 were censored.

Luminal A subtype generally has better survival compared to the other molecular subtypes. The underestimation on this subgroup could have been affected by the unknown Ki‐67 value for majority of patients. Introduction of Ki‐67 testing into clinical practice was delayed in Japan until around 2011, including KUH, because most clinicians have been skeptical of the significance of Ki‐67 expression until that time. Hence, unknown Ki‐67 status was 70.6% in this study. Currently, a value of >20 for Ki‐67 differentiates an ER positive and PR positive tumor into a Luminal B subtype versus Luminal A subtype; if Ki‐67 is unknown, such will be categorized as Luminal A.

The underestimation in long‐term survival of patients with histologic grade 3 tumors can be caused by the lack of representation of this group in the study validation population. It can likewise be due to the variations in the treatment approaches or some other prognostic variable differences among the study population and the tool development population.

PREDICT also underestimated the impact of breast‐conserving surgery, no systemic therapy, ETx only, and 10 years ETx on survival. These are usually utilized in patients with lower‐risk tumors, thus, explains the higher survival. Since PREDICT was based on a population gathered from 1999 to 2003, patients who had these treatments were probably underrepresented. Nowadays, substantially more patients are being treated with breast‐conserving techniques and endocrine therapy.

The key strengths of this study are the large population size and the nearly complete data on nonrequired yet important variables on PREDICT, such as mode of detection and HER2 status. To our knowledge, this is the first validation study on PREDICT tool version 2.2 that was based on the Japanese population. A limitation of this study is the deficiency of Ki‐67 data on majority of the validation population. Testing for Ki‐67 was not routine in KUH until the latter months of 2010, as well as throughout Japan. Ki‐67 serves a significant role in breast cancer prognosis,^32^ and the missing Ki‐67 data on majority of patients may have affected the results. Another weakness of this study is the heavy censoring in the validation population. This might have affected the observed survival estimates based on the Kaplan–Meier curve.

## 5 CONCLUSION

PREDICT tool accurately estimated the 5‐ and 10‐year OS rates in the entire Japanese validation population. Hence, PREDICT may be considered as a valid prognostication tool for breast cancer patients in Japan. However, caution should be used for interpretation of the 5‐year OS outcomes in patients that are ≥65 years old, and also for the 10‐year OS outcomes in patients that are ≥65 years old, those with histologic grade 3 and Luminal A tumors, and in those considering ETx or no systemic treatment.

## DISCLOSURE

MK has received honorariums as a speaker or consultant/advisory role from Chugai Pharmaceutical Co. (Tokyo, Japan). The other authors declare that they have no conflict of interest.

## Data Availability

Data sharing is not applicable to this study.
